# Impact of visual mouth image presentation on speech perception in noise in normal hearing subjects and cochlear implant users

**DOI:** 10.1371/journal.pone.0357983

**Published:** 2026-09-11

**Authors:** Anna Maria Schneider, Timo Stöver, Tobias Weissgerber

**Affiliations:** 1 Audiological Acoustics, Department of Otorhinolaryngology, University Hospital, Goethe University Frankfurt, Frankfurt am Main, Germany; 2 Department of Otorhinolaryngology, University Hospital, Goethe University Frankfurt, Frankfurt am Main, Germany; LSU Health Shreveport, UNITED STATES OF AMERICA

## Abstract

This study investigated the impact of visual speech cues on speech perception in noise and subjective listening effort in cochlear implant (CI) users compared with normal-hearing (NH) individuals. Furthermore, the study compared visual information presented either by a computer-generated avatar or by video recordings of a real speaker. Thirteen NH participants and twelve bilateral CI users completed the Oldenburg Sentence Test (OLSA) in six conditions: auditory‑only, visual‑only, and audiovisual presentations using either video recordings (female speaker) or a computer‑animated avatar (male speaker). Speech reception thresholds (SRTs) in noise and subjective listening effort (LE) were measured. Visual‑only lipreading performance and self‑assessed lipreading skills were also recorded. Video‑based audiovisual presentation significantly improved speech perception compared with audio‑only presentation (NH: 1.6 dB; CI: 3.5 dB). CI users demonstrated substantially higher lipreading scores than NH participants (51% vs. 28%), and lipreading performance strongly correlated with self‑reported ability. Despite SRT improvements, audiovisual presentation yielded only marginal reductions in listening effort for CI users and none for NH participants. In contrast, the computer‑animated avatar provided no measurable audiovisual benefit for speech perception or listening effort in either group. Real‑speaker video recordings considerably enhance audiovisual speech perception in both NH individuals and CI users, particularly benefiting CI listeners. However, reductions in listening effort were minimal, underscoring the dissociation between speech perception and perceived listening effort. Incorporating audiovisual measures may improve ecological validity in clinical assessment of CI outcomes.

## Introduction

Communication is an important part of daily lives and contributes significantly to social well-being [1]. In adverse communication conditions with different talkers and noise and/or reverberation, speech perception is particularly challenging for individuals with hearing impairment [2]. Challenges in communication can result in increased stress and social isolation. In such situations hearing impaired individuals will use coping mechanisms such as turning one´s ear toward the sound source or even avoiding settings that are perceived as too noisy [3]. Another compensation mechanism is trying to read the mouth image (i.e., “lipreading”) [4]. While hearing impaired individuals are oftentimes trained in reading mouth images, even normal hearing (NH) individuals can benefit from audiovisually presented speech [5]. The absence of visual information provided by the mouth image is particularly eminent in the context of medical protective measures, such as the wearing of face masks (e.g., in medical institutions or during the Covid-19 pandemic). It was shown that the absence of visual information is the main reason for worse speech perception when the speaker is wearing a face mask [6], whereas the sound attenuation of the face mask was only of minor impact [7,8].

Consequently, the access to the mouth image is of utmost importance in individuals with hearing impairment, especially in cohorts with severe degrees of hearing loss or users of cochlear implants (CIs, [9]). CIs have been implemented as a highly effective treatment for cases in which even the most powerful hearing aid has been unable to provide sufficient speech perception [10]. CI users often reach speech perception scores in quiet close to results of NH individuals. Furthermore, CI implantation can also significantly improve other measures like listening effort and quality of life as well as cognitive abilities including attention, working memory, and executive functions [11,12]. Additionally, patients reported improvements not only in hearing and verbal communication but also positive changes respecting additional factors such as a reduction of tinnitus, depression, cognitive decline, somatization disorders and loneliness [13].

However, CI users still demonstrate a massively degraded performance in speech perception in noise and reverberation compared to NH individuals [2,14–17]. Clinical speech tests to evaluate hearing performance of CI users are typically conducted under free-field conditions in quiet or with only one noise source. In everyday listening, acoustic scenes are more complex. Furthermore, many individuals with hearing impairment report that they find speech perception in noisy environments to be fatiguing requiring a considerable amount of listening effort [18]. Particularly in complex listening situations in everyday life, reading the mouth image could potentially improve speech perception and reduce listening effort. This effect is not considered in clinical setups so far.

The objective of the present study is to compare the impact of visual information on speech perception in noise and on subjective listening effort between CI users and NH individuals. Furthermore, results for different presentation methods using either a computer-generated avatar or video recordings of a real person were evaluated.

## Materials and methods

### Participants

The study included 25 participants (13 NH, 12 with bilateral CI). The NH group consisted of six male and seven female participants (mean age: 26.3 ± 3.9 years, range: 21.1–35.1 years). Inclusion criteria for NH participants were based on pure-tone audiometry (i.e., air conduction thresholds better than 25 dB HL for the test frequencies 0.25 kHz, 0.5 kHz, 1 kHz, 2 kHz, 4 kHz, 8 kHz). Furthermore, none of the NH participants reported on having tinnitus.

The CI group consisted of four male and eight female participants (mean age: 60.4 ± 10.2 years, range: 40.2–76.0 years). CI participants were bilaterally provided with cochlear implants from the manufacturers Cochlear (Sydney, Australia) and MED-EL (Innsbruck, Austria) and used their own sound processors with everyday programming and volume settings during testing. CI participants had at least one year hearing experience with their secondly implanted ear (mean: 6.3 ± 3.8 years). Prior to the study tests CI participants were asked to self-assess their lipreading skills on a scale from zero to ten (0: no lipreading abilities, 10: perfect lipreading abilities). Demographic details of the CI subjects are shown in Table 1.

**Table 1:** Demography of CI users including age, type of implant and sound processors, daily sound processor wearing time, hearing experience with CIs, bilaterally aided Freiburg monosyllable scores (FMS), and self-assessed lipreading skills (0 = none to 10 = perfect).

ID	Age [yrs]	Type of Implant r / l	Sound Processors r / l	Daily wearing time r / l [hrs]	Hearing experience r / l [yrs]	FMS [%]	subjective lipreading skills
1	76.0	CI422/CI512	CP1000/CP1000	16.1 / 16.1	10.7 / 11.9	95	4
2	63.5	CI532/CI422	CP1000/CP1000	14.9 / 15.2	6.3 / 10.3	95	3
3	69.7	Concerto/Concerto	Sonnet 2/Sonnet2	13.7 / 13.7	11.7 / 10.6	80	3
4	64.1	Concerto/Concerto	Opus 2/Opus 2	n.a./ n.a.	10.5 / 11.5	95	5
5	53.2	Synchrony/Synchrony	Sonnet/Sonnet	13.0 / n.a.	6.9 / 7.8	90	7
6	59.4	CI532/CI532	CP1110/CP1110	14.6 / 14.7	7.0 / 4.0	80	5
7	66.2	CI612/CI512	CP1000/CP1000	12.4 / 2.2	3.4 / 4.4	95	6
8	48.8	Synchrony2/Synchrony2	Sonnet2/Sonnet2	14.6 / 14.4	1.9 / 3.2	75	10
9	62.0	Synchrony2/Synchrony2	Sonnet/Sonnet2	17.9 / 16.6	4.5 / 3.5	80	1
10	72.2	Pulsar/Sonata	Sonnet2/Sonnet2	14.8 / 14.8	12.7 / 15.3	65	7
11	40.1	Synchrony2/Synchrony	Sonnet2/Sonnet	15.2 / 14.7	3.7 / 4.3	90	8
12	49.7	Synchrony2/Synchrony2	Rondo3/Rondo3	13.6 / 13.8	2.4 / 1.2	85	6

All test subjects had normal vision or corrected vision (either glasses or contact lenses). No further assessments of visual function were performed as part of the study. For the recruitment of participants with cochlear implants, patient records were reviewed to ensure that there were no indications of cognitive impairment (e.g., dementia) documented. No additional assessment of cognitive performance was conducted prior to the administration of the study tests.

The study was approved by local Ethics Committee (No. 2021−408). Participants provided written informed consent. Patients were recruited and data was collected between 01/01/2023 and 30/09/2023.

### Test setup

Study tests were conducted in an anechoic chamber (size: 4.10 m x 2.60 m x 2.10 m, length x width x height). Participants were seated on a chair viewing on a 22” screen (distance to test subject: 1.2m, placed at a height of 1.5m) which was used for the presentation of the mouth image. A loudspeaker at frontal position (0°, distance to test subject: 1.2m) was used for sound presentation.

Speech perception in noise was tested using the Oldenburg sentence test (OLSA, [19]). The OLSA is a matrix test consisting of sentences arranged in different test lists. Every sentence includes the same five types of words (name, verb, number, adjective, item). For every test list each word gets randomly chosen out of ten options. The options vary for each test list. This makes the sentences semantically correct, but not meaningful and unpredictable in terms of content. Therefore, the test can be repeated as many times as desired with the same person without any substantial learning effects after the participant has practiced a few lists. Speech reception threshold (SRT) for 50% correct word perception was adaptively measured in unmodulated noise matching the long-term frequency spectrum of the test sentences (“OLnoise”) with a fixed level of 60 dB SPL. Speech and noise were presented co-located from the same loudspeaker. The initial signal-to-noise ratio (SNR) was 0 dB for the NH participants and +5 dB for the CI participants.

Participants had to indicate the perceived words on a touch screen monitor. Furthermore, the participants were asked after each sentence to indicate their subjective listening effort (LE) on a categorical scale (Effort Scale Categorical Units, ESCU) from “no effort” (category: 1 ESCU) to “extremely effortful” (category: 13 ESCU) with the additional option to choose “only noise” as category 14 ESCU [20]. The SRT was adaptively measured using 20 OLSA trials. During these 20 trials, the subjective LE was recorded concurrently. Afterwards, 10 additional OLSA trials using SNRs between −10 dB and +20 dB SNR were conducted to assess the subjective LE within an extended SNR range. Subjective LE at 0 dB SNR was calculated using a linear regression of all LE measures.

The OLSA test was conducted in a version with a female speaker [21] as well as in a version with male speaker [19]. In total, six different test conditions were defined: (1) OLSA female auditory, (2) OLSA female audiovisual, (3) OLSA female visual, (4) OLSA male visual, (5) OLSA male auditory, (6) OLSA male audiovisual. After four OLSA runs for training purposes including the male/female auditory and audiovisual test conditions, SRTs and subjective LE for the six test conditions were assessed in a randomized order.

**Fig 1:**
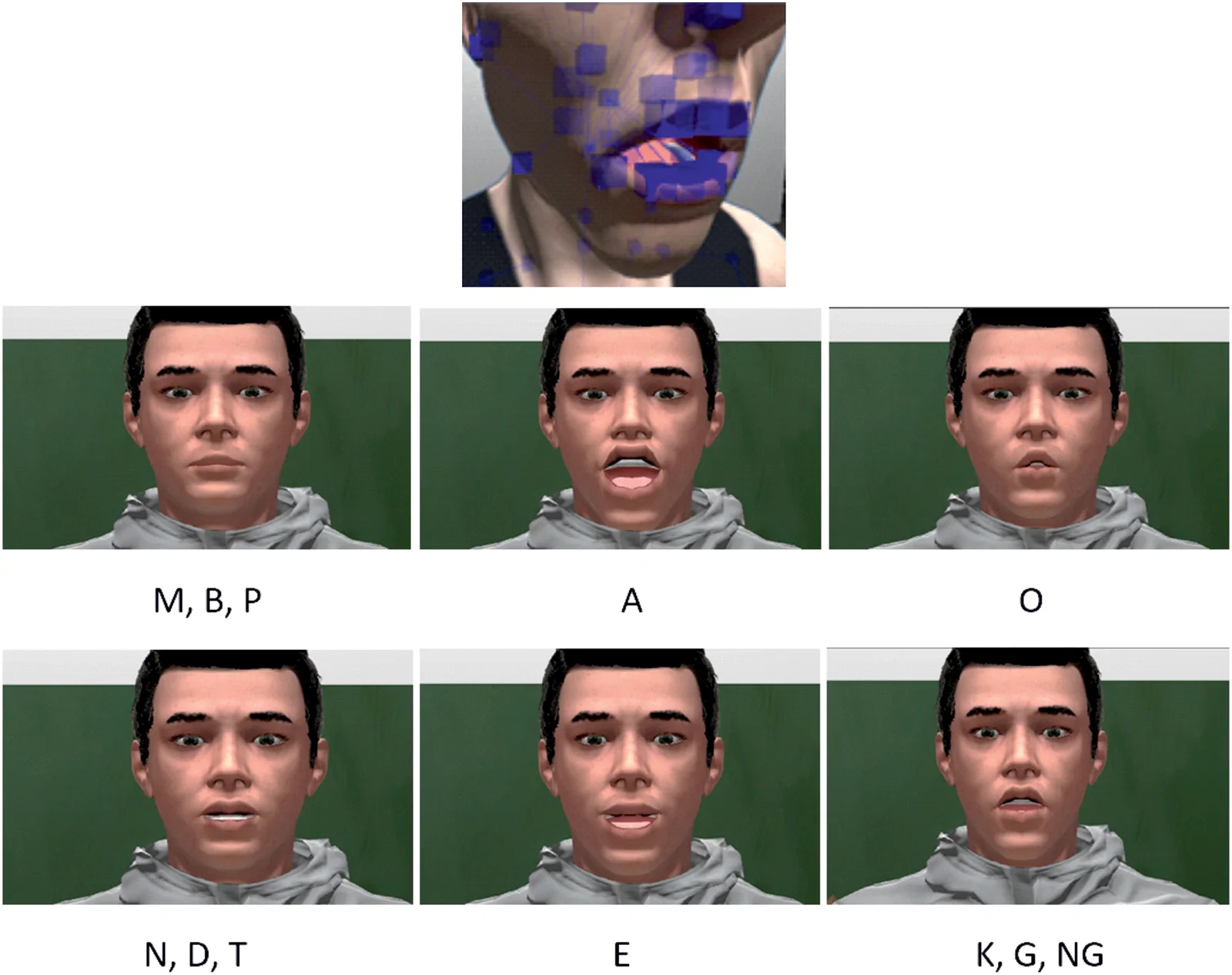
Computer-animated avatar showing the used facial points (upper) and examples for visemes / mouth images created for different phonemes (lower).

For the visual and audiovisual parts of the test, two different visualization methods were incorporated. For the female version, video recordings were used [22]. For the male OLSA, lip-synchronized facial animations were created using a 3D-modeled avatar (Unity Multipurpose Avatar, UMA Steering Group) in Software Unity (Unity Technologies, San Francisco, USA). The individual facial points of the avatar were assembled into face parameter groups (see Fig 1) which were controlled by UMA expression player: jaw movement (up/down, forward/backward, left/right), mouth movement (left/right), mouth narrowing, mouth expression (smile/frown, left side), mouth expression (smile/frown, right side), left lower lip (up/down), right lower lip (up/down), left upper lip (up/down), right upper lip (up/down), tongue protrusion (out/in), tongue movement (left/right, up/down), tongue width (wide/narrow), and tongue rolling.

Individual facial parameter sets (i.e., control data for the face parameters in UMA expression player) were created for each viseme according to [23] in order to build a sequence of visemes for each OLSA sentence. Smooth transitions between consecutive visemes were achieved through interpolated blending, resulting in more natural mouth movements.

The sequence of visemes was assessed by phonetic transcription of the OLSA audio material using the web-based BAS service [24,25]. For temporal synchronization of audio and visual material the temporal progression of the phonemes was extracted with the software Praat (Institute of Phonetic Sciences, University of Amsterdam, Netherlands) and transformed into keyframes in the animation timeline of the Unity software.

### Statistics

Statistical analyses of differences in SRT were performed using nonparametric tests. Between‑group differences were evaluated using the Mann–Whitney U test. Within‑group differences were examined using the Wilcoxon signed‑rank test. Both were selected due to their robustness under non‑normal distributional assumptions. A p-value < 0.05 was considered as significant. Bonferroni correction was used in case of multiple comparisons. SPSS Statistics 29 (IBM, Armonik, New York, USA) was used to analyze the test results.

## Results

### Speech perception and listening effort in noise

**Fig 2:**
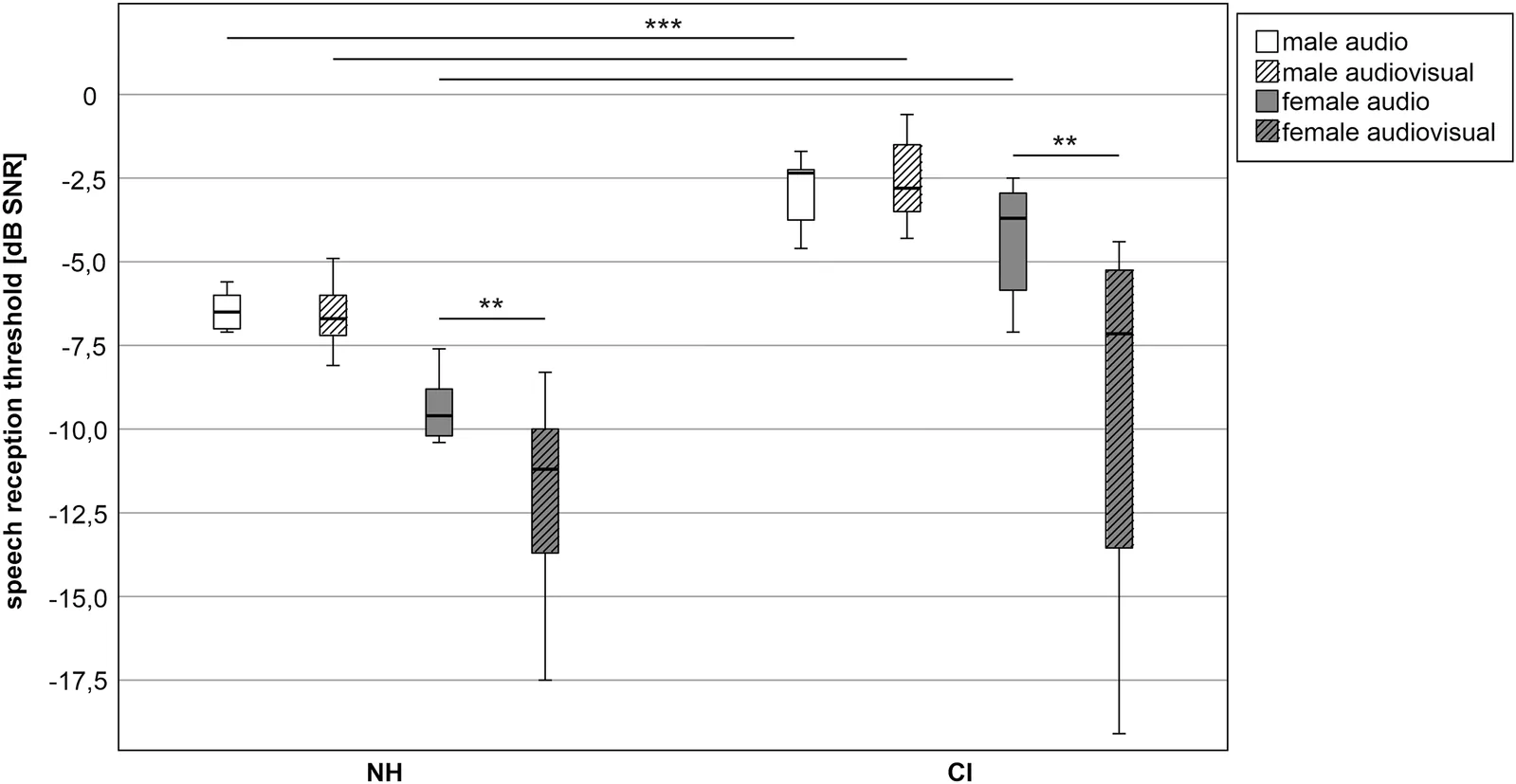
Speech reception thresholds in noise [dB SNR] for NH (n = 13) and CI (n = 12) participants for the test conditions male speaker auditive presentation, male speaker audiovisual (avatar) presentation, female speaker auditive presentation, and female speaker audiovisual (video recording) presentation.

**Fig 3:**
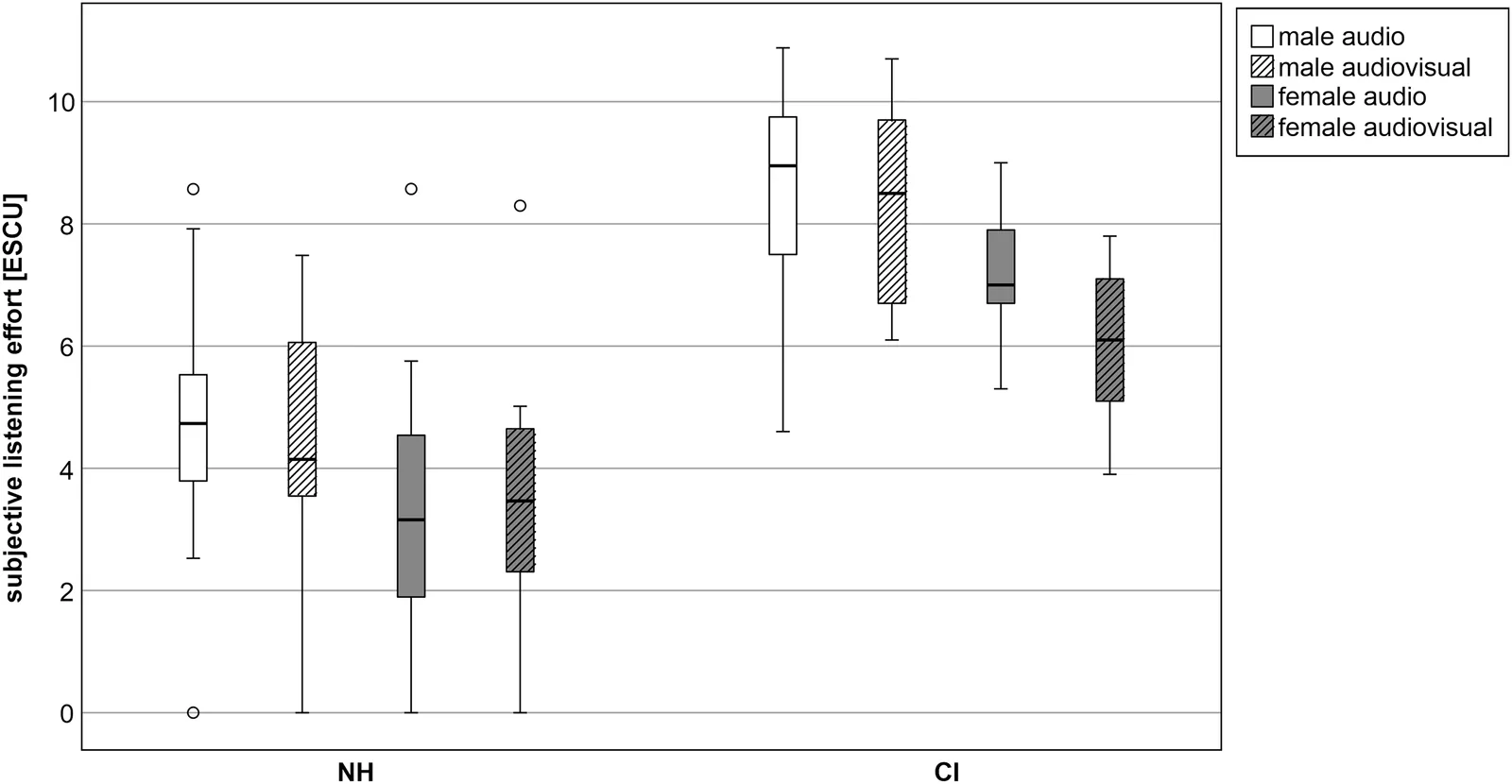
Subjective listening effort at 0 dB SNR [ESCU] for NH (n = 13) and CI (n = 12) participants for the test conditions male speaker auditive presentation, male speaker audiovisual (avatar) presentation, female speaker auditive presentation, and female speaker audiovisual (video recording) presentation.

Results of the SRT measurements in noise depending on test condition and subject group are shown in Fig 2. Results of the subjective rating of listening effort at 0 dB SNR depending on test condition and subject group are shown in Fig 3.

### Impact of speaker type

There was a significant effect of speaker type (male vs. female) on SRTs and subjective listening effort. For auditory only stimulus presentation, SRTs in the female speaker condition were significantly lower than in the male speaker condition for both subject groups (NH: −6.5 vs. −9.6 dB; Z = ‑3.183, p = 0.003; CI: −2.4 vs. −3.7 dB; Z = ‑3.062, p = 0.006). For audiovisual stimulus presentation, SRTs in the female speaker condition were also significantly lower than in the male speaker condition for both subject groups (NH: −6.7 vs. −11.2 dB; Z = ‑3.181, p = 0.003; CI: −2.8 vs. −7.2 dB; Z = ‑3.059, p = 0.006). For auditory only stimulus presentation, listening effort in the female speaker condition was significantly less effortful than in the male speaker condition for both subject groups (NH: 4.7 vs. 3.2 ESCU; Z = ‑2.981, p = 0.009; CI: 9.0 vs. 7.0 ESCU; Z = ‑2.432, p = 0.045). For audiovisual stimulus presentation, listening effort in the female speaker condition was only in the CI group significantly lower than in the male speaker condition (NH: 4.1 vs. 3.5 ESCU; Z = ‑2.223, p = 0.078; CI: 8.5 vs. 6.1 ESCU; Z = ‑2.983, p = 0.009).

### Impact of subject group

In three out of four test conditions, the NH group achieved significantly lower SRTs than the CI group: In the auditory only condition, a significant SRT difference was found for the male speaker (4.2 dB; U = 0, Z = −4.252, p = 0.004) and the female speaker (5.9 dB; U = 0, Z = −4.248, p = 0.004). In audiovisual condition, only the difference for male avatar condition of 3.9 dB was statistically significant (U = 0, Z = −4.248, p = 0.004). In contrast, the SRT difference for the female video condition of 4.1 dB was not statistically significant (U = 42.0, Z = −1.959, p = 0.208).

In all four test conditions, the NH group reported significantly lower ratings of listening effort than the CI group (male audio: U = 13.0, Z = −3.536; male audiovisual: U = 10.0, Z = −3.699; female audio: U = 12.0, Z = −3.591; female audiovisual: U = 17.0, Z = −3.318; all p-values = 0.004). Depending on test condition, NH participants rated listening effort between “very little effort” (3.2 ESCU) and “little effort” (4.7 ESCU). On the contrary, CI users entered higher rating between “moderate effort” (6.1 ESCU) and “significant effort” (9.0 ESCU).

### Impact of visual presentation modality (avatar vs. video recording)

Neither the NH group nor the CI group could benefit from the mouth image of the animated avatar in the male audiovisual condition to improve SRTs. On the other hand, in the female audiovisual condition using video recordings, SRTs in the NH group significantly improved by 1.6 dB compared to auditory only presentation (Z = ‑3.112, p = 0.006). The group of CI users showed an even higher significant improvement of 3.5 dB by reading the mouth image (Z = ‑3.061, p = 0.006).

Neither the NH group nor the CI group could benefit from the mouth image of the animated avatar in the male audiovisual condition to reduce listening effort. The audiovisual presentation of the video recording of the female OLSA did not reduce listening effort in the NH group. Listening effort in the CI group was reduced by 0.9 ESCU by reading from the mouth of the female video recording compared to auditory only presentation. However, this small difference became insignificant when the Bonferroni correction for multiple comparisons was applied (Z = ‑2.394, p = 0.051, p_uncorrected_ = 0.017).

### Lipreading abilities

**Fig 4:**
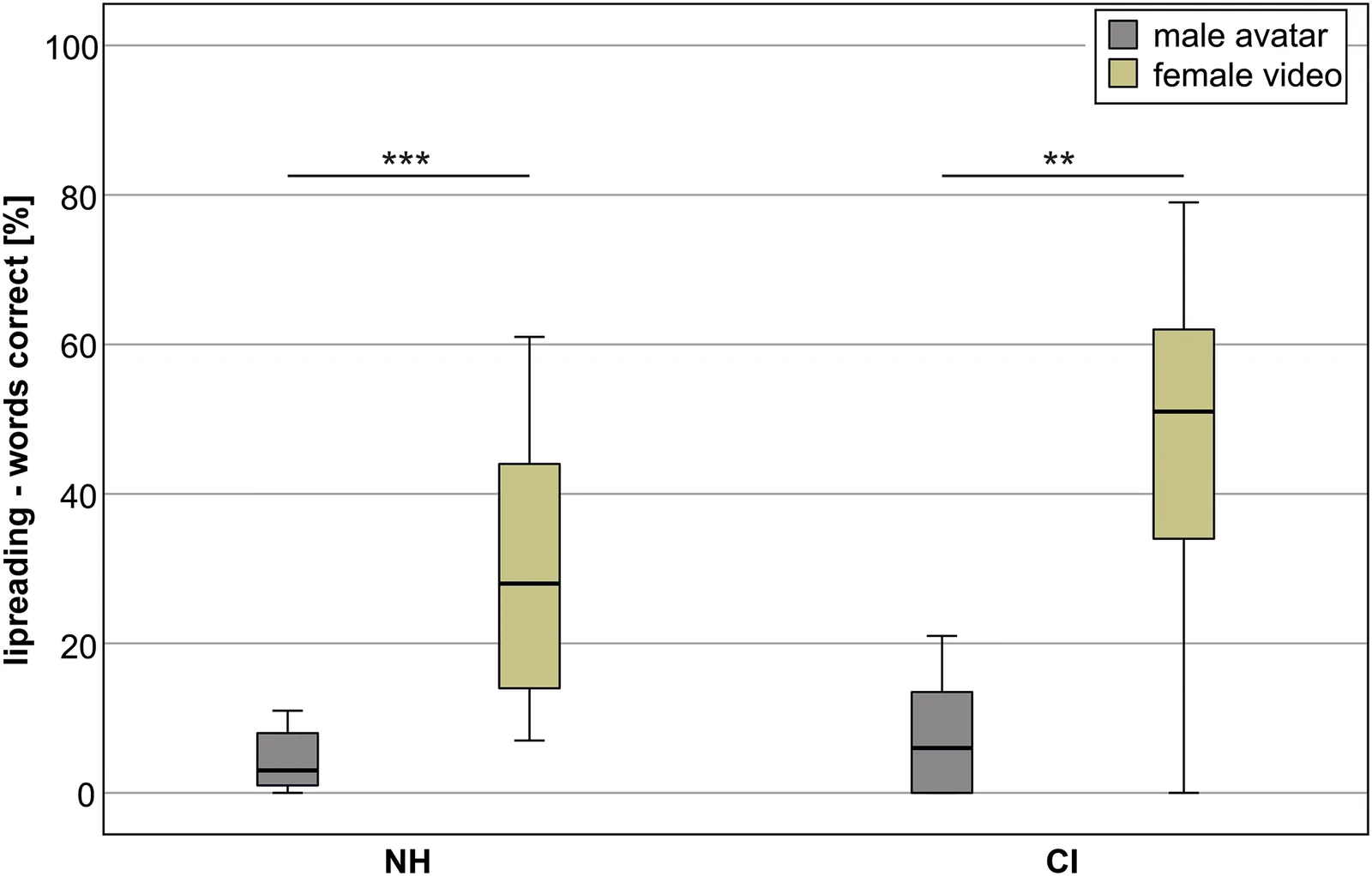
Lip reading ability for visually presented information of male animated avatar (grey) and female OLSA video recordings (dark yellow) for NH (n = 13) and CI (n = 12) participants.

Results of the ability to read the mouth image without any acoustic information depending on visual test condition (avatar vs. video recording) and subject group are shown in Fig 4.

There was a significant impact of visual presentation modality (avatar vs. video recording) on visual word recognition rate in both subject groups. While almost no lipreading was possible in the avatar condition (NH group: 3%, CI group: 6% words correct), lipreading scores for female video recordings improved significantly to 28% (NH group, Z = ‑3.182, p = 0.001) and 51% (CI group, Z = ‑2.936, p = 0.003). A comparison of the lipreading skills between both subject groups in the female video recording condition revealed 23 percentage points superior lipreading proficiency in the CI group (U = 36, Z = ‑2.286, p = 0.044), whereas no group difference was found in the avatar condition.

**Fig 5:**
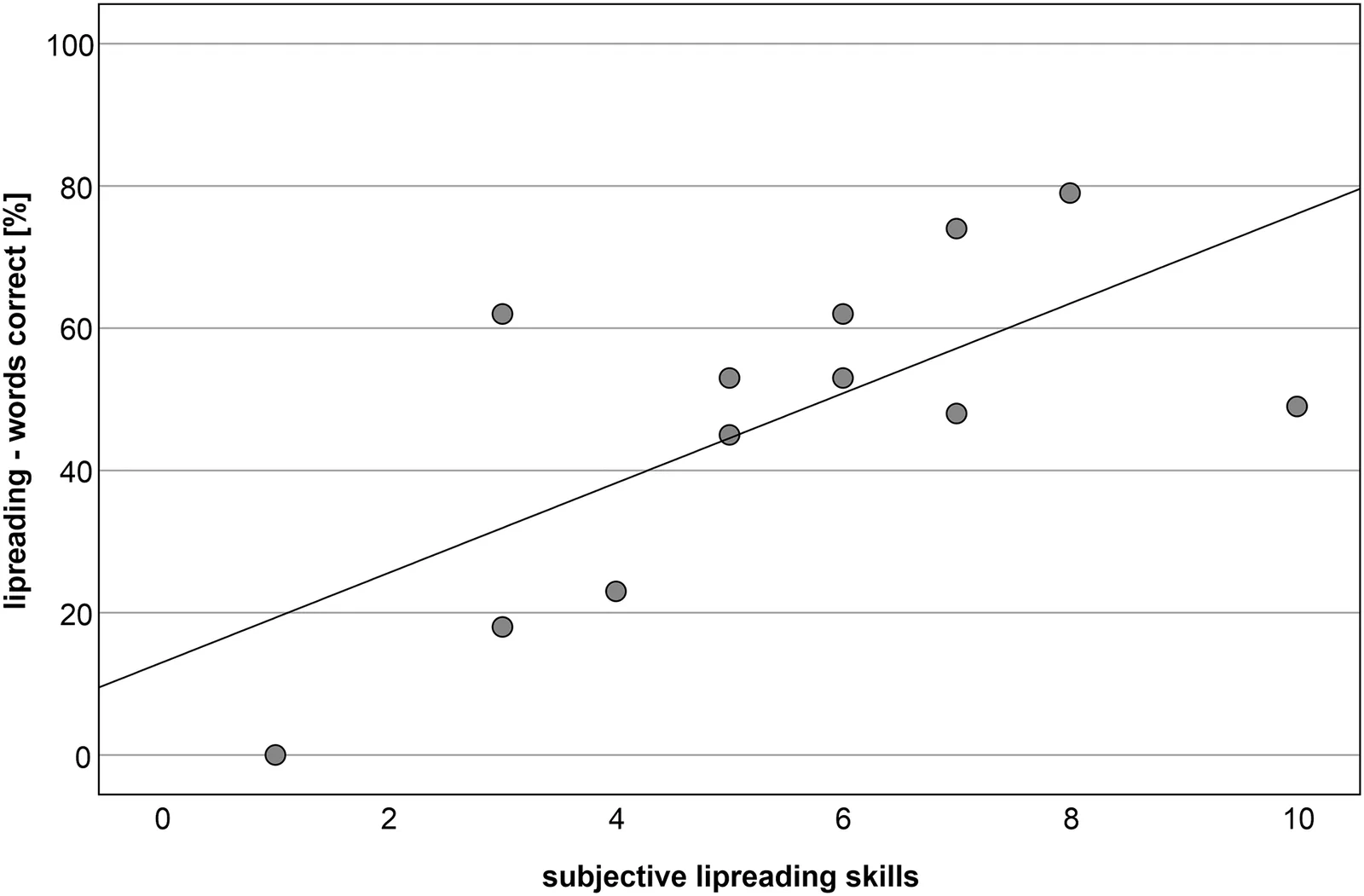
Correlation between word recognition rate based on the mouth image (video recording, visual only condition) and self-assessed lipreading skills for CI (n = 12) participants.

Fig 5 shows the relation between subjectively rated lipreading skills and word recognition rate in the visual only test condition with video recordings (female OLSA) in the CI group. There was a significant correlation between subjective lipreading skills and word recognition rate in the test condition with video recordings (r = 0.671; p = 0.017). However, no correlation was found between subjective lipreading skills and SRT in the audiovisual test conditions.

## Discussion

### Male vs. female OLSA in acoustic-only condition

The present findings corroborate previously reported differences between the male and female OLSA speakers. The mean SRTs measured for the female OLSA in the NH group (−9.6 dB) closely match the reference values published by Wagener et al. [21] of −9.4 dB. Likewise, the SRTs obtained in the male OLSA for the NH group (mean SRT: −6.5 dB) correspond well with previously reported data (‑7.1 dB, [21]). The consistently poorer SRT in the male speaker condition across subject groups is likely attributable to speaker‑specific acoustic characteristics, such as higher speaking rate and lower fundamental frequency in the male speaker. The larger vocal cavity area in the female speaker compared to the male speaker is also related to differences in speech perception in noise [26].

Consistent with these findings, subjective listening effort was also lower for the female OLSA in both groups (NH group: 1.5 ESCU, CI group: 2 ESCU). This is in line with results by Schulte et al. [27] reporting that in the male OLSA a higher SNR is necessary to achieve the same listening effort.

### Effect of visual information (video recordings) on speech perception

CI users achieved significantly higher lipreading scores than NH participants (51% vs. 28%) in the visual-only condition, a finding consistent with previous results [28]. A strong correlation between self‑reported lipreading ability and visual wordrecognition scores further supports the notion that CI users develop highly individual visual compensation strategies, which substantially contribute to their lipreading performance (range: 0–80%). Consequently, and in line with earlier studies [22,29], both NH participants and CI users showed improved SRTs in noise when visual information from video recordings of a real speaker was provided. Although the improvement of 1.6 dB observed in the NH group was somewhat smaller than previously reported values (3–5 dB), such deviations may reflect interindividual differences in lipreading ability and the degree of training of different study cohorts [30]. Consistent with previous evidence CI users rely more strongly on visual cues than NH to compensate for degraded auditory input [4,31–33]. The CI group demonstrated a substantially larger visual benefit of 3.5 dB. Sönnichsen and coworkers [6] reported a mean visual benefit of 4.5 dB for CI users and 2.7 dB for NH listeners when using the same OLSA video recordings as those employed in the present study.

When interpreting the results, however, it is important to note that half of the CI users had a visual word recognition rate of more than 50%. A consistent visual word recognition rate (i.e., lipreading ability) of more than 50% would theoretically (for an infinite number of trials) lead to an audiovisual SRT of minus infinity, since the acoustic part has no more impact on the measured SRT at all. Therefore, individual CI subjects with very advanced lipreading achieved SRT improvements higher than 10 dB. Therefore, in clinical routine, audiovisual speech‑perception measurements at fixed SNRs may be more suitable than SRT‑based assessments.

### Effect of visual information (video recordings) on subjective listening effort

Despite clear improvements in SRT, the audiovisual condition resulted in only marginal reductions in listening effort for CI users and no improvement for NH participants. In the CI group, listening effort was only reduced in the audiovisual test condition using video recordings (female OLSA) by 1 ESCU, but only significant without Bonferroni correction. Even subjects with very advanced lipreadings skills had only improvements in listening effort between −0,5 (i.e., even higher effort) and 2.2 ESCU. This contradicts the considerable average SRT improvement of 3.5 dB and suggests that better speech perception does not necessarily translate into reduced subjective effort. Although visual speech information improves recognition performance, it can also increase the cognitive load required when simultaneously processing degraded auditory information and visual speech cues in the audiovisual condition [34]. This effect was found across all age groups with the strongest effects observed in older adults.

CI participants in the present study reported LE ratings that were 3–4 ESCU higher than those of the NH group. Similarly, Abdel‑Latif and coworkers found approximately 3 ESCU higher LE ratings in CI users compared with NH participants at identical SNRs [35].

### Impact of age on speech perception, audiovisual integration and listening effort

When considering age as a factor in audiovisual speech perception, literature presents mixed findings. Rohner et al. reported a decline in speech‑reading ability with increasing age [8]. Other studies also found that younger NH [36,37] and CI [36] individuals achieve better visual‑only scores than older participants. In contrast, Tye‑Murray et al. showed that the overall benefit derived from combining auditory and visual information remains stable across adulthood [38], indicating that age-related differences may affect visual‑only speech reading but not necessarily audiovisual integration. Dias and Schvartz-Leyzac reported that NH and CI users rely more on multisensory integration as they age [33]. This effect of improved multisensory additivity with increasing age was also found by Dias and coworkers [37].

Previous studies also confirmed that speech perception in noise declined with increased age in normal hearing adults (with hearing thresholds matched to young adults, [39]) and in CI users [40]. Furthermore, negative age effects on listening effort in auditory‑only speech‑in‑noise conditions were found, even though the tested age groups achieved identical wordrecognition scores [41] or when age-related variance in speech recognition was partialled out [42]. An age effect on listening effort was also documented when listening to speech signals with degraded intelligibility [43].

It is important to note that all CI users in the present study were older than the participants in the NH group (U = 0, Z = −4.243, p < 0.001). Notably, seven out of twelve CI users were over 60 years of age. This age difference may also have contributed to the observed differences in audiovisual gain and to the generally higher subjective listening effort in the CI group across both audio-only and audiovisual test conditions compared with the NH group. On the one hand, CI users had greater lipreading experience which was reflected in better results in the visual only condition. On the other hand, age-related changes in audiovisual integration and speech perception may have influenced listening effort and performance compared to the younger NH group independently of hearing loss and cochlear implant use.

### Lipreading and audiovisual gain using a computer-animated avatar

A key finding of the present study is that the animated avatar failed to provide measurable benefit in audiovisual speech perception. Neither NH participants nor CI users showed improvements in SRTs when the avatar was presented, and lipreading performance in visual only condition was near floor level for both groups. This indicates that the implemented avatar, despite phoneme-based synchronization, does not yet approximate the articulatory detail or naturalness required for effective visual speech perception. This stands in marked contrast to the substantial audiovisual benefit obtained from video recordings shown in the present study and previous studies. The current findings therefore emphasize that the avatar cannot be considered an adequate visual substitute for real facial speech cues. One critical aspect in the presented approach could be the transition between visemes. To avoid visible glitches and to achieve a smooth transition between visemes, blending (i.e., interpolation) between the facial parameters of each viseme was conducted. The blending parameters were optimized to obtain the subjectively best visual outcome. However, it cannot be ruled out that blurred coarticulation contributed to the poor outcome. More advanced animation techniques may offer improved outcomes.

Schreitmüller and coworkers used a computer-animation (“talking head”) and found audio-visual gains comparable to those obtained by video recordings [36]. Furthermore, word recognition rate in a visual only condition using the talking head (NH:30%, CI:47%) was comparable to the findings of the present study using video recordings.

A different promising approach involves avatars whose facial animations are derived directly from real video data, enabling high‑fidelity modeling of mouth movements and coarticulation patterns [44]. Such data‑driven methods may offer a more naturalistic visual speech signal and could therefore serve as an effective alternative to live-action recordings in future research and clinical applications.

### Study limitations

In this study, a relatively simple approach was used to animate the avatar. It is conceivable that more advanced animation techniques could improve speech perception by providing more accurate and interpretable mouth movements. Therefore, the lack of audiovisual benefit observed for the computer‑animated avatar in the present work should not be generalized, as more sophisticated avatar models may yield different outcomes.

Furthermore, it should be noted that the CI and NH groups were not age‑matched, with the CI participants being older on average than the NH participants. However, comparison between the two groups was not the primary objective of the present study.

## Conclusion

Video recordings of a real speaker can enhance speech perception compared with audio-only presentation. This beneficial effect of visual speech cues was more pronounced in CI users but was also evident in NH individuals. In contrast, NH reported no and CI users only marginal reduction of subjective listening effort. Therefore, depending on the clinical question, incorporating measures of listening effort into routine testing may be advantageous to capture these distinct aspects of performance.

Since visual cues are often available in everyday communication, audiovisual testing may represent a meaningful extension of clinical outcome assessment, offering a more ecologically valid measure of patients’ real‑life communication abilities.
